# Evaluation of *Chlorella vulgaris* biosorption capacity for phosphate and nitrate removal from wastewater

**DOI:** 10.1038/s41598-023-50748-3

**Published:** 2024-01-09

**Authors:** Amany A. Asaad, Amany S. Amer

**Affiliations:** 1 Inorganic Department, Central Laboratory for Environmental Quality Monitoring (CLEQM), National Water Research Center (NWRC), Cairo, Egypt; 2 Biology and Environmental Indicators Department, Central Laboratory for Environmental Quality Monitoring (CLEQM), National Water Research Center (NWRC), Cairo, Egypt

**Keywords:** Biochemistry, Biotechnology, Chemical biology, Environmental sciences, Natural hazards

## Abstract

High wastewater production rates during the past few decades are mostly attributable to anthropogenic activities. The main components leading to the nutrient enrichment of natural water bodies are such as nitrogen, phosphorus, and other minerals. The main focus of this research was to assess the ability of using *Chlorella vulgaris* algae, a potent and environmentally benign material, to eliminate phosphate and nitrate ions from wastewater. FTIR results showed that the biologically active molecules that facilitate the binding of phosphate and nitrate ions unto the *C. vulgaris* are C=C and N–H amid. The ideal equilibrium time for adsorption was 24 h with an optimum pH of 7 and the mass ratio of algae and different anions concentration was 80%. Freundlich isotherm model was the best-fitted isotherm. Moreover, the results of the experiment fit more closely with the pseudo-second-order kinetic model than other models. Elovich kinetic model data for both ions showed that the adsorption rate was much higher than the desorption rate. The growing popularity of biosorbents in treating wastewater has led to an improvement in their affordability and availability, and *C. vulgaris* may now represent an environmentally friendly choice from an environmental, and economic standpoint.

## Introduction

Wastewater contamination of nutrients might become a more serious issue if it is disposed of improperly. This can have a detrimental influence on the improvement of the environment and people’s quality of life in addition to having significant negative effects on agricultural development^1^. Both phosphate and ammonia can offer serious health concerns to people involving the possibility of illnesses such methemoglobinemia in young children despite their presence in wastewater effluents in low amounts ammonia can also be detrimental to aquatic species including fish^2^. Repeated wastewater disposal in lack of sufficient and suitable treatment may cause serious environmental issues. Diverse watery organisms that convert sunlight to energy include algal cells in their structure. They either have one cell (microalgae) or many (macro algae). Algae don’t have branches, origins, or leaflets like higher plants do^3^. The organisms of the with a size between a few and hundreds of micrometres are known as microalgae. Microalgae may double their total number of cells in 24 h and have a 12-day exponential growth cycle with a doubling time of only 35 h^4^. They efficiently absorb nutrients into the body cells and reproduce every few hours in suitable autotrophic or mixed trophic settings^5–7^. Microalgae and macroalgae possess innate capabilities to efficiently accumulate nutrients, such as nitrogen (N) and phosphorus (P), from diverse aquatic environments. This natural nutrient accumulation enables them to synthesize a diverse array of bioactive compounds, including pigments, carbohydrates, proteins, and lipids, thanks to their wide-ranging physiological and biochemical attributes. These bioactive compounds hold significant potential for various commercial applications^8,9^.

A number of investigations are now being done on the utilisation of microalgae cultural systems to treat industrial, municipal, and agricultural wastewaters^10–12^. Both nourishment encourage the spread of living things like algae, which in turn supply habitat as well as food for fish, shellfish, and other small water-dwelling creatures. In Malaysia, where a lot of citizens rely on reservoirs as their only source of water for use in drinking and cooking; and elevated amounts of such nutrients in the water causing contamination^13^. This might potentially have harmful effect even in low-level contents^14^. A few advantages of using algae are that they are at lower cost, results in valuable biomass which is benefited in many ways and better quality of renewable effluent water^15^. In addition, The metabolic processes and development of algae is greatly affected by phosphorous in addition to nitrogen. By phosphorylation and nitrogen intake, algae take out elements from a growing environment. The patterns of nutrient uptake can vary depending on the presence of nutrients^7^. Algae species have demonstrated variable efficacy as well as effectiveness in removing nitrogen and phosphorus out of different wastewater streams due to their varying physiology and morphologies. This is in part depends on nitrogen-to-phosphorous (N/P) ratio, which is vital for the formation of biomass in a variety of algae and the efficient and simultaneous intake of nutrients^16^. This N/P ratio enables developing algae in wastewater to consistently show a quicker intake of nitrogen compared to phosphorous. Due to their fast rate of development and excellent adaptation for different wastewater streams, strains within the genus *C. vulgaris* have been widely employed and discovered to be ideal for growth in wastewater^17^. The novelty of this study is the evaluation of a wastewater treatment method using environmentally friendly, highly productive and cost-effective materials (microalgae *C. vulgaris*) in removing nutrients and can then be used as biofertilizer after the treatment process^18^.

## Materials and methods

### Algae source

**Figure 1:**
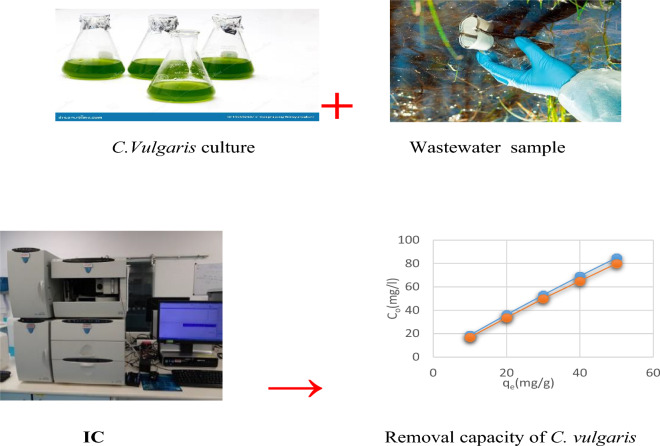
Experimental analysis of wastewater sample.

*Chlorella vulgaris* was obtained from the Algae Unit Research, National Research Centre, Egypt. It was used without any chemical pre-treatment for sorption experimentations (Fig. 1).

### Preparation of nitrate and phosphate stock solution

A stock solution of 1000 mg/L nitrate and phosphate ions was prepared using KH_2_PO_4_ and NaNO_3_ salts (merck). A series of different initial concentrations (10–50 mg/l) of nitrate and phosphate working solutions were prepared. The pH of each solution was adjusted to (6.5–8) using 0.1N (HCl) and 0.1N (NaOH) and the pH was estimated using a pH meter (WTW Inolab pH meter).

### Estimation of nitrate and phosphate

The analysis of nitrate and phosphate ions was carried out using an ICs-5000 ion chromatography system equipped with a conductivity detector the separation process was conducted on an AS19 column (2 × 250 mm 4 µm) with protection from a guard column ag19 (2 × 50 mm 4 µm). The column temperature was maintained at a constant 35 °C throughout the procedure the mobile phase employed was potassium hydroxide at a flow rate of 1.00 ml min^−1^ and it was generated by an eluent generator (EGC 500). Elution was achieved through a gradient pump starting with an initial KOH concentration of 10 mM for 0.5 min, the concentration was then gradually increased to 25 mM over 15.5 min followed by a rapid increase to 35 mM within 0.1 min, this concentration was held at 350 Mm for 8 min and then adjusted back to the initial composition stabilizing for an additional 10 min. the suppressor used was an ADRS 600 4 mm in size operating in reciprocal mode at 38 Ma. The injection volume was set at 2 µl and the entire analysis run lasted for 35 min. All ions were identified by comparing retention times with standard solutions. Quantification was based on externally calibrated peak areas and the concentration range was 5–20 mg L^−1^. All standards were obtained from HACH.

### Bio-sorption parameters affecting nitrate and phosphate removal

#### Effect of pH

The bio-sorption of nitrate and phosphate by *C. vulgaris* was studied at different pH values from 6.5 to 8. The required pH values were modified by 0.1N HCl and 0.1N NaOH solutions^19^.

#### Effect of bio-sorbent mass

Different mass ratios (10%, 20%, 40%, 60%, 80%) of *C. vulgaris* were added to nitrate and phosphate concentrations of 20 mg L^−1^ at normal pH to determine the optimal mass ratio affecting the adsorption process^20^.

#### Effect of initial concentration

In the bio-sorption trials, 20 ml of nitrate and phosphate were shaken in different concentrations (10, 20, 30, 40, and 50 mg L^−1^) with live algae in the ratio (1: 4) at 140 rpm for 24 h. using a shaker. Filtration processes were done using Wattman no. 42 filter paper and the filtrates were taken and analysed using ion chromatography (ICs500) to determine the amounts of NO_3_ and PO_4_ after adsorption. The amount of NO_3_ and PO_4_ ions adsorbed by *C. vulgaris* was derived from the following equations:

1 $$q_{e} = \left( {C_{o} - C_{e} } \right)\frac{V}{W}$$

2 $$\% \;{\text{ions}}\;{\text{removal}} = \frac{{\left( {C_{o} - C_{e} } \right)}}{{C_{o} }} \times 100$$

where C_o_ is the starting point of nitrate and phosphate ions (mg L^−1^), C_e_ is the final balance number of nitrate and phosphate ions (mg L^−1^), V is the total amount of nitrate and phosphate solution (L), and W is the weight of the bio-sorbent selected for adsorption^5,21^.

#### Effect of contact time

Different contact times (24, 48, and 60 h) were chosen to determine the optimal time affecting ion uptake on *C. vulgaris* algae at room temperature at 25 °C and pH 7. The mass ratio of the bio-sorbents (80%), shake speed 140 rpm and the starting nitrate and phosphate ions concentration (20 mg L^−1^) stayed stable in all circumstances.

### Adsorption isotherms and kinetics studies

Isotherm and kinetic studies were performed under conditions of 80% adsorbent mass ratio, contact time 24 h, and pH 7 at room temperature 25 °C. The mechanism of nitrate and phosphate adsorption on *C. vulgaris* algae was explained using isotherm models, to calculate the adsorbents performance and investigate their mass transfer mechanisms Kinetic models were applied, physiosorption is covered by pseudo-first order, chemisorption is covered by pseudo-second order, and the mass and surface diffusion are predicted by the Elovich kinetic model.

#### Isothermal models

In adsorbate-adsorbent systems, Eqs. 3 and 4 use the Langmuir isotherm to illustrate monolayer adsorption, while Eqs. 5 use the Freundlich isotherm to show adsorption occuring on heterogeneous surfaces. In addition, Eq. 6 takes into account the Temkin isotherm model, which states that as the amount of adsorbent surface occupancy increases, all molecules' adsorption energies should drop linearly.

Langmuir adsorption equations:

3 $$\frac{{C_{e} }}{{q_{e} }} = \frac{1}{{q_{m} K_{L} }} + \frac{{C_{e} }}{{q_{m} }}$$

4 $$R_{L} = \frac{1}{{1 + \left( {1 + K_{L} C_{o} } \right)}}$$

where q_e_ is the adsorption capacity at equilibrium, C_e_ represents the equilibrium concentrations of nitrate and phosphate, q_max_ is the maximum adsorption capacity at equilibrium, and K_L_ is Langmuir constant which indicates the adsorption energy. C_o_ is the initial concentration of adsorbate and R_L_ explains the adsorption preference of this isotherm and indicates whether the adsorption is irreversible if R_L_ = 0, linear if R_L_ = 1, or unfavourable if R_L_ > 1^22^.

Freundlich adsorption equation:

5 $$\ln q_{e} = \ln K_{F} + \frac{1}{n}\ln C_{e}$$

where n is the adsorption intensity and K_F_ is the Freundlich isotherm constant used to assess the adsorption capacity of *C. vulgaris*. The heterogeneity parameter is 1/n, and a smaller value of 1/n denotes a more heterogeneous medium^23^.

Timken adsorption equation:

6 $${\text{q}}_{{{\text{e}} }} =\upbeta _{{{\text{T}} }} \ln {\text{K}}_{{{\text{T}} }} +\upbeta _{{\text{T }}} \ln {\text{C}}_{{{\text{e}} }}$$

where at equilibrium, q_e_ is the amount of nitrate and phosphate adsorbed (in mg/g), and C_e_ is the concentration of nitrate and phosphate in solution (in mg/L). In the formula for the heat of adsorption constant is = RT/b, T is for the absolute temperature (K), b for the Temkin constant (J/mol), R for the gas constant (8.314 J/mol K), and K for the Temkin isotherm constant (L/g)^24^.

#### Kinetic models

In order to determine the adsorption rate and mechanism, three models (pseudo-first order, pseudo-second order, and Elovich model) were examined.

(a) Pseudo-first-order equation

7 $$\log (q_{e} - q_{t} ) = \log q_{e} - \left( {\frac{{K_{1} }}{2.303}} \right)t$$

where qe is the quantity of nitrate and phosphate ions attracted by *C. vulgaris* at balance in (mg/g), qt is the amount at any given time in (mg/g), and K_1_ is the pseudo-first-order model’s kinetics rate constant (min^−1^)^25^.

(b) Pseudo-second-order equation

8 $$\frac{t}{{q_{t} }} = \frac{1}{{K_{2 } q_{e}^{2} }} + \frac{t}{{q_{e} }}$$

where K_2_ is the kinetics steady-state rate of the pseudo-second-order model (g mg^−1^ min^−1^).

(c) Elovich model equation

The nonlinear and straightforward form of the Elovich kinetic model is expressed by (Eqs. 9, 10) respectively^26^.

9 $$q_{t} =\upbeta \ln (\upalpha \upbeta {\text{t}})$$

10 $$q_{t} = \frac{1}{\upbeta }\ln (\upalpha \upbeta {\text{t}}) + \frac{1}{\upbeta }\ln {\text{t}}$$

where q_t_ (mg/g) is the adsorbate quantity at time t, α is a chemisorption rate constant and β is a constant that represents the amplitude of surface coverage. α and β can be calculated from the relation between their slope and intercept by plotting q_t_ versus lnt.

## Results and discussions

### Characterization of *C. vulgaris*

#### SEM micrograph

**Figure 2:**
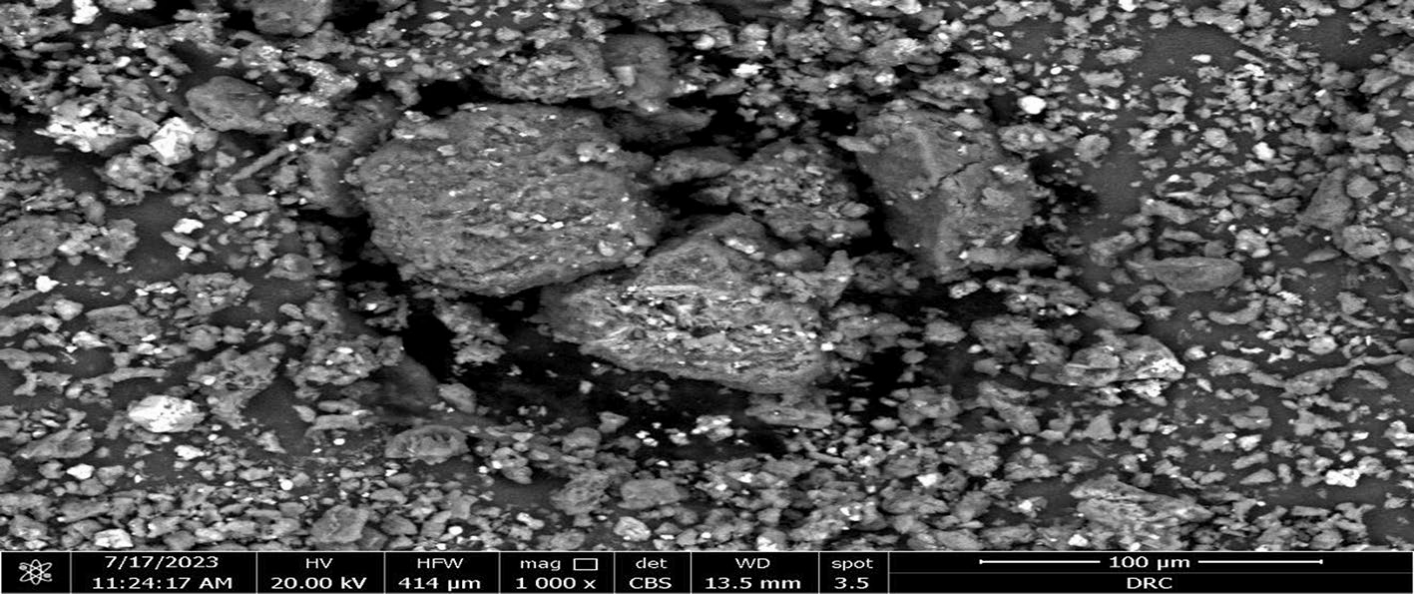
Scanning Electron Microscope of *C. vulgaris* algae.

For absolute morphological examinations, the dried sample of *C. vulgarise* algae was imaged using Scanning Electron Microscopy (SEM). Analysis of the morphology of green microalgae reveals uneven particle frameworks, and the outer layer of the cells is wrinkled, porous, and has more fractures, all of which could offer a significant outer area and a significant number of spots of activity for both anions adsorption (Fig. 2).

#### FTIR spectral analysis

**Figure 3:**
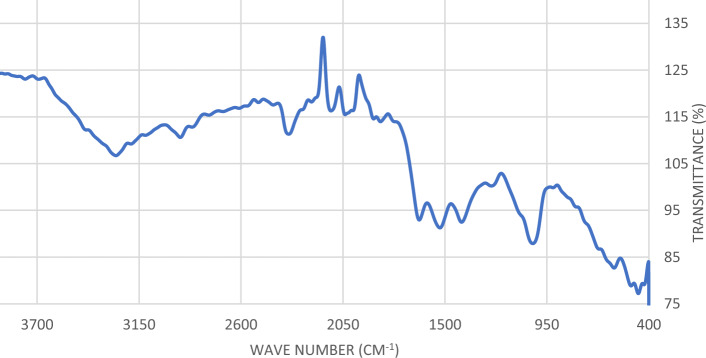
Infra-red spectra of *C. vulgaris* algae before phosphate and nitrate removal.

**Figure 4:**
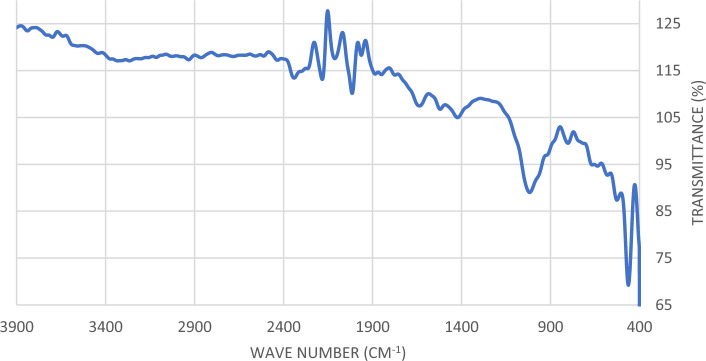
Infra-red spectra of *C. vulgaris* algae after phosphate and nitrate removal.

With respect to modifications in the vibrational frequencies, FTIR analysis was used to differentiate between the effective compounds found in *C. vulgaris* before and following phosphate and nitrate ion adsorption. As shown in (Table 1) where the measured effective groups and the wavenumber ranges were recorded. The band at 3275.07 cm^−1^ related to (N–H) amid^27^ which was responsible for the binding of phosphate and nitrate ions unto the *C. vulgaris*. It was found that bands at1638.74 cm^−1^ and 1527.28 cm^−1^ were related to C=C and δ (N–H) amid respectively which were responsible for the effective removal of ions as shown in (Figs. 3, 4)^28,29^.

**Table 1:** Spectral data before and after adsorption for *C. vulgaris*.

Wave number range (cm^−1^)	Before adsorption	After adsorption	Assignment
3029–3639	3275.07	Disappeared	N–H amid band
2809–3012	2927.67	Disappeared	C=C stretch
1583–1709	1638.05	1636.84	C=C stretch
1481–1585	1527.28	Disappeared	Amid δ(N–H) band
1357–1423	1410.18	1422.80	C=C stretch
980–1072	1026.35	1017.17	Polysaccharides C–O–C

### Effect of biosorption parameters

#### pH

**Figure 5:**
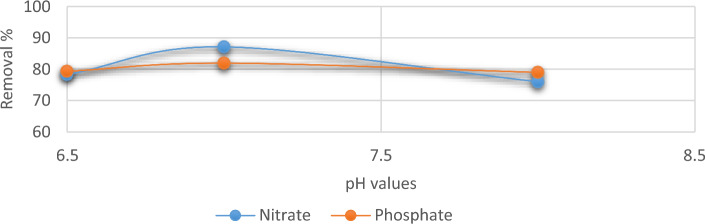
Removal efficiency of phosphate and nitrate at different pH using *C. vulgaris*.

With an opposing attraction according to the relative pH of the liquid form, phosphate and nitrate ions attach to the binding the out layer of biosorbents^30^. In the current instance, the removal of nitrates and phosphates with *C. vulgaris* was most favored in the pH range of 6.5–8.0. Figure 5 shows that the upper limit of adsorption, for nitrate was (17.42 mg g^−1^) and the greatest carrying capacity for phosphate was (16.13 mg g^−1^). The elimination of both anions was slightly less at pH 8 than it was at pH 7. Because of the intensive competition with OH ions in the anions solution at the higher pH, the lower reduction in phosphate and nitrate at the higher pH level can lead to a poor affinity of ions towards the adsorption sites of *C. vulgaris* algae^31^. At lower and higher pH values, the sorption efficiency was significantly reduced. The majority of the sites are negatively charged at pH less than 7, which increased the repulsion effect and reduced the number of anions that could be adsorbate^32,33^.

#### Bio-sorbent dose

**Figure 6:**
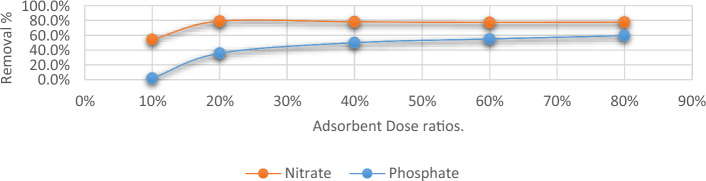
Removal efficiency of phosphate and nitrate at different doses of *C. vulgaris*.

As depicted in Fig. 6, the concentration of two adsorbed anions increased as the dose of *C. vulgaris*’s variously concentrated solutions was increased from 10 to 80% (20 mg L^−1^). As a result of the increased adsorbent dose, there were more responsive spots and outer layer patches accessible to the ions to bind to^34,35^. As the dosage was raised, the amount of phosphate and nitrate that *C. vulgaris* was able to adsorb per unit mass decreased due to an excess of accessible sorption sites after the anions had completely been adsorbed into the solution. The decreasing trend may possibly be related to the concentration gradient’s splitting effect^36^.

#### Contact time

**Figure 7:**
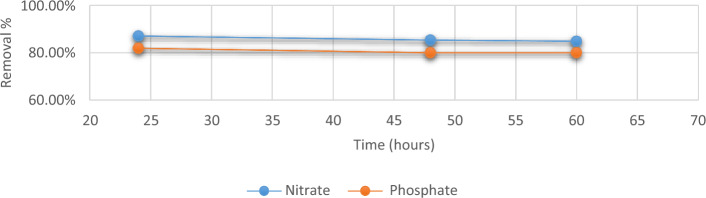
Removal efficiency of phosphate and nitrate at different times using *C. vulgaris*.

There is a swift and substantial initial absorption of phosphates and nitrates from wastewater within the initial 24-h period is depicted in the kinetic profile (Fig. 7) of the process while at 60 h of contact time, the gradient begins to decline. As the anions are adsorbed onto the *Chlorella* surface, as the adsorption process continues, the number of available adsorption sites decreases, leading to a gradual flattening of the slope in the adsorption rate lowers, explaining why the initial uptake occurs more quickly^37,38^. All subsequent experiments were conducted at 24 h contact time.

#### Phosphates and nitrates initial concentrations

**Figure 8:**
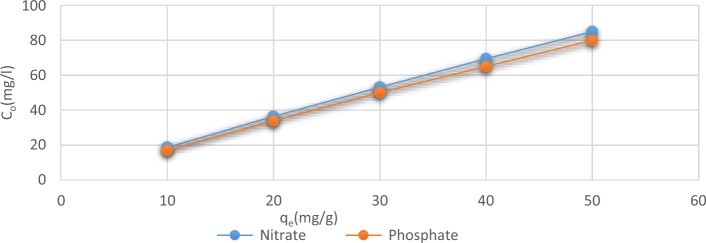
Removal capacity of *C. vulgaris* at different concentrations of phosphate and nitrate ions.

It proved that since the original phosphate and nitrate ions content grew, so did the *C. vulgaris* algae’s ability for absorption. The test applied a variety of anion contents (between 10 and 50 mg L^−1^). The starting concentration of 10 mg L^−1^ was having the lowest adsorb observed, while the concentration of 50 mg L^−1^ was the greatest adsorption. The initially measured phosphate concentration increased, while the percentage of anions removed dropped (Fig. 8). Because the original amounts of phosphate and nitrate ions increasing, there was a rise in the moles ratio of these ions to the algae’s reachable outer layer, which resulted in a reduced clearance percentage^39^. Since the biosorbent material dose was fixed in the current case, the number of anions spots of adsorption on the algae remained constant lowering the percentage of phosphate and nitrate ions clearance by raising their initial concentration^30^.

## Adsorption isotherms

**Figure 9:**
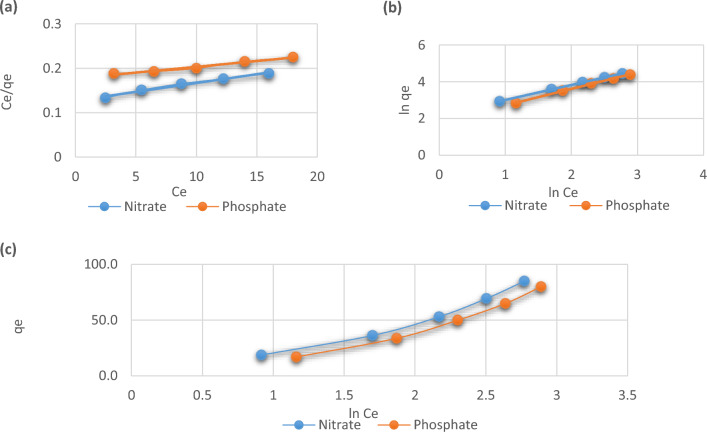
(**a**) Langmuir, (**b**) Freundlich, and (**c**) Timken isotherms for phosphate and nitrate adsorption unto *C. vulgaris*.

For adsorption study, the adsorption isotherms have significance because they express the link within the absorbent quantity and its accumulation on the exterior of the adsorbent and calculate the adsorbent’s capability for biosorption. The experimental data were fitted utilising the linear forms of the Langmuir, Freundlich, and Timken isotherms in order to assess the performance of *C. vulgaris* algae at 25 °C; the resulting plots depicted in Fig. 9a–c. Table 2 displays the *C. vulgaris* algae’s phosphate and nitrate adsorption isotherm ratios which were identified using the isotherm plots. The data from experiments that have the highest correlation coefficient values are compatible with the Freundlich isotherm. A heterogeneous adsorption procedure might be utilised to clarify the manner in which the results on phosphate and nitrate adsorption conform to Freundlich isotherm brought on by multilayer adsorption as well as the exponential distribution of adsorbent active sites and their energies towards the phosphate and nitrate ions onto the surface of the algae^40^. The maximum adsorption capacity (Q_max_) of phosphate and nitrate calculated by the function was 250 and 384.6 mg g^−1^ respectively. The value of 1/n = 1 and 0.89 for nitrate and phosphate respectively, while n = 1.2 and 1.1 indicate that the sorption of phosphate and nitrate ions unto *C. vulgaris* is favorable and the value of R^2^ = 1 and 0.99 for nitrate and phosphate ions respectively indicating that *C. vulgaris* was a favorable biosorbent to remove phosphate and nitrate ions from aqueous solutions^41^.

**Table 2:** Isotherms results of adsorbed ions unto *C. vulgaris*.

Adsorption isotherms	Adsorption parameters	Phosphate ions	Nitrate ions
Langmuir	Q_max_ (mg/g)	250	384.6
	K_L_ (L/mg)	0.03	0.015
	R_L_	0.76	0.87
	R^2^	0.98	0.98
Freundlich	K_F_(mg/g)	6.13	8.96
	1/n	0.89	0.82
	n	1.11	1.23
	R^2^	0.99	1.00
Temkin	β_T_	35.99	35.05
	K_T_ (L/mg)	2.23	1.67
	R^2^	0.97	0.96

## Kinetic models

**Figure 10:**
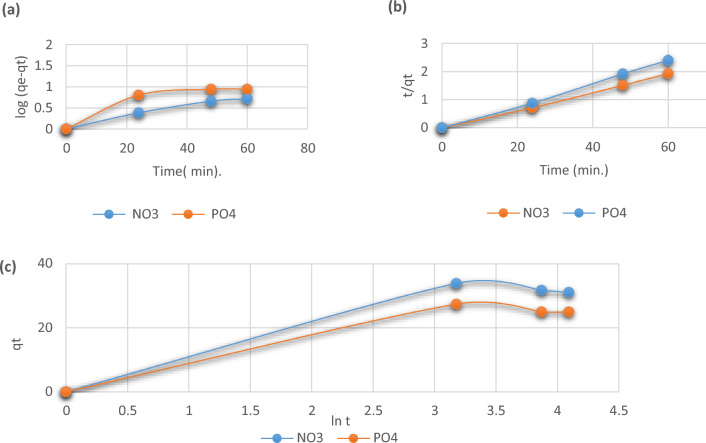
(**a**) Pseudo-first-order, (**b**) Pseudo-second-order, and (**c**) Elovich kinetic models for phosphate and nitrate adsorption unto *C. vulgaris*.

Using pseudo-first-order, pseudo-second-order, and Elovich kinetic models, the kinetic adsorption of phosphate and nitrate ions with *C. vulgaris* was investigated. The relationship between the number of empty sites and the number of occupied adsorbent sorption sites is made clearer by the pseudo-first-order kinetic model^42^. Figure 10a shows the linear relation between ln (q_e_ − q_t_) against time and then rate constant k_1_ is measured. According to the pseudo-second-order kinetic model depicted in Fig. 10b, a relationship between the adsorbent’s capacity for adsorption and time can be formed. By calculating the slope and intercept of the linear plot of t/q_t_ against time^43^, one may get the rate constants k_2_ and q_e_. Similar to how the Elovich kinetic model is used, the plot of q_t_ against ln t yields a straight line from which the values of α and b may be calculated based on the slope and intercept, as shown in Fig. 10c. With regard to the other models, the pseudo-second-order kinetic model provided an excellent match to the results of the experiment in which R^2^ > 0.99 (near the unity) and also gives higher q_e_ values for both phosphate and nitrate ions. The Elovich kinetic model data of both ions showed that the adsorption rate (α, mg/g.min) is much higher than the desorption rate (β, g/mg)^44^. All results of the applied kinetic models are shown in Table 3.

**Table 3:** Kinetic results of adsorbed ions unto *C. vulgaris*.

Kinetic models	Phosphate ions	Nitrate ions
Pseudo-first-order	R^2^ = 0.800	R^2^ = 0.970
	q_e_ = 1.47 mg/g	q_e_ = 1.09 mg/g
	K_1_ = 0.035 min^−1^	K_1_ = 0.028 min^−1^
Pseudo-second-order	R^2^ = 0.998	R^2^ = 0.0.995
	q_e_ = 24.75 mg/g	q_e_ = 31.05 mg/g
	K_2_ = 0.05 g/mg min	K_2_ = 0.04 g/mg min
Elovich	R^2^ = 0.91	R^2^ = 0.92
	q_t_ = 19.44 mg/g	q_t_ = 24.17 mg/g
	α = 7.79 mg/g min^−1^	α = 9.68 mg/g min^−1^
	β = 0.12 g/mg	β = 0.15 g/mg

## Treatment application with real wastewater sample

**Figure 11:**
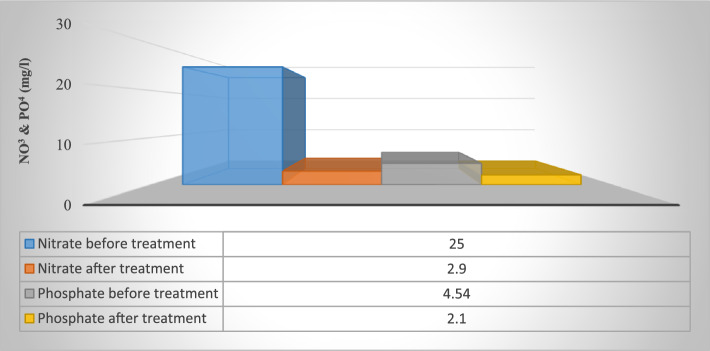
Analysis of wastewater samples prior to and post phosphate and nitrate adsorption unto *C. vulgaris*.

The remediation process which is done by algae as a microorganism is known as phytoremediation. One of the algal species which take place in this bioremediation technique is *C. vulgais*^15^. Although it is difficult to compare the effect of algal culture in wastewater treatment, various studies have demonstrated that algal formation can support nutrient removal in wastewater^8^ The selected agricultural wastewater sample was provided from the El-Rahawy drain known with its detectable concentrations of ammonia and phosphate. The wastewater sample was stored in a plastic container at 4 °C. The optimum conditions of pH, contact time and concentrations were considered. In the Sample, the phosphate ions concentration was 4.54 mg L^−1^ and for nitrate ions concentration was 25 mg L^−1^. The removal efficiency of *C. vulgaris* after the adsorption of PO_4_ and NO_3_ was 53.74% and 88.4% respectively (Fig. 11).

## Conclusion

The purpose of the research was to determine the removal efficiency of *C. vulgaris* for both phosphate and nitrate ions which is decreased with increasing the initial concentration. Different effective parameters pH, adsorbent doses, contact time, and different initial concentrations were measured to select the optimum conditions under which the adsorption occurred. The results of the experiment showed that the optimum equilibrium time for adsorption was 24 h with an optimum pH of 7 and the mass ratio of algae and different anions concentration was 80%. According to FTIR investigation, the groups with functional properties like carboxylic, amid, and carbonyl (C=C and N–H amid), may be implicated in the reaction, ion complexation among anions and *C. vulgaris* may happen in the biosorption process. The impacts of all the variables on the anions adsorption were carefully assessed and the outcomes of the experiment fit the Freundlich adsorption isotherm model well. This shows that heterogeneity adsorption has the highest rates of 250 and 384.6 mg g^−1^ phosphate and nitrate, respectively. The values of R^2^ = 1 and 0.99 for nitrate and phosphate ions respectively. Indicating the chemical adsorption process, the pseudo-second order kinetic model fit the data well than other models with R^2^ > 0.99 and higher q_e_ values of 24.75 and 31.08 mg g^−1^ for both phosphate and nitrate ions, respectively. Elovich kinetic model data for both ions showed that the adsorption rate (α, mg/g min) was much higher than the desorption rate (β, g/mg). The experimental findings suggest that *C. vulgaris* may be used as a biosorbent for phosphate and nitrate ions in wastewater. Thus, it can be concluded that *C. vulgaris* can be effectively employed for the removal of phosphate and nitrate from the surface water.

### Recommendation

According to results of *C. vulgaris* removal capacity for nutrients, the future object is it can be use as alternate source for agricultural sectors in reducing the usage of harmful chemical fertilizers.

## Data Availability

The datasets generated during and/or analyzed during the current study are available from the corresponding author on reasonable request.
